# OLDER ADULT KIDNEY TRANSPLANT RECIPIENTS: THE LIVED EXPERIENCE OF ADAPTATION AND INTEGRATION

**DOI:** 10.1093/geroni/igz038.510

**Published:** 2019-11-08

**Authors:** Laura L Kimberly, Denise Burnette, Ellen Lukens, Bruce Gelb

**Affiliations:** 1 Columbia University, New York, New York, United States; 2 Virginia Commonwealth University, Richmond, Virginia, United States; 3 NYU Langone Health, New York, New York, United States

## Abstract

As the prevalence of end-stage renal disease increases in the United States, a growing number of adults aged 65 and over are receiving kidney transplants. While older adult recipients tend to fare well from a clinical standpoint, far less is known about their psychosocial wellbeing following transplantation. This study seeks to better understand the lived experience of older adult kidney transplant recipients, focusing on the ‘liminal’ period of adaptation following organ transplantation that has been reported elsewhere in the literature. Applying the hermeneutic phenomenology of philosopher Paul Ricoeur, the study explores the lived experience of 10 deceased donor kidney transplant recipients aged 65 and over. Guided by Ricoeur’s conceptual approach to identity as constituted through two forms, idem and ipse, preliminary findings suggest that despite expressing some distress around the ‘strangeness’ of integrating part of another into oneself (disruption of the idem sense of self), participants also constructed powerful narratives of resilience and coping that were rooted in the continuity of a deeply held ipse sense of identity over the life course. In particular, participants emphasized their ability to overcome adversity as an anchor of their ipse sense of self that enabled them to navigate the idem corporeal changes of transplantation. Moreover, they described kidney transplantation as a form of liberation, ultimately restoring their idem sense of self that had been profoundly disrupted by ‘machine life’ (time spent on dialysis). These findings will have significant implications for ensuring the provision of optimal support to older adult kidney transplant recipients.

